# The bigger the threat, the longer the gaze? A cross-cultural study of Somalis and Czechs

**DOI:** 10.3389/fpsyg.2023.1234593

**Published:** 2023-09-27

**Authors:** Iveta Štolhoferová, Daniel Frynta, Markéta Janovcová, Veronika Rudolfová, Hassan Sh Abdirahman Elmi, Kateřina Rexová, Daniel Alex Berti, David Král, David Sommer, Eva Landová, Petra Frýdlová

**Affiliations:** ^1^Department of Zoology, Faculty of Science, Charles University, Prague, Czechia; ^2^Department of Biology, Faculty of Education, Amoud University, Borama, Somalia

**Keywords:** Africa, eye-tracking, snake, Snake detection theory, spontaneous attention

## Abstract

High fear reaction, preferential attention, or fast detection are only a few of the specific responses which snakes evoke in humans. Previous research has shown that these responses are shared amongst several distinct cultures suggesting the evolutionary origin of the response. However, populations from sub-Saharan Africa have been largely missing in experimental research focused on this issue. In this paper, we focus on the effect of snake threat display on human spontaneous attention. We performed an eye-tracking experiment with participants from Somaliland and the Czechia and investigated whether human attention is swayed towards snakes in a threatening posture. Seventy-one Somalis and 71 Czechs were tested; the samples were matched for gender and comparable in age structure and education level. We also investigated the effect of snake morphotype as snakes differ in their threat display. We found that snakes in a threatening posture were indeed gazed upon more than snakes in a relaxed (non-threatening) posture. Further, we found a large effect of snake morphotype as this was especially prominent in cobras, less in vipers, and mostly non-significant in other morphotypes. Finally, despite highly different cultural and environmental backgrounds, the overall pattern of reaction towards snakes was similar in Somalis and Czechs supporting the evolutionary origin of the phenomenon. We concluded that human attention is preferentially directed towards snakes, especially cobras and vipers, in threatening postures.

## 1. Introduction

Snakes seem to evoke a number of specific responses in humans, including a high fear reaction, preferential attention, and fast detection (Öhman and Mineka, 2003; Okon-Singer et al., 2011; Soares et al., 2014; Kawai and Qiu, 2020; Landová et al., 2020; Jensen and Caine, 2021). Each of these phenomena has been previously studied in detail and is described in its own terms and hypotheses. Taken altogether, however, the human mind seems to be specifically equipped to react to snakes in a certain manner (Isbell, 2006, 2009). Further, there are several compelling pieces of evidence that this reaction is at least partially innate (Tierney and Connolly, 2013; Kawai, 2019). First, it is shared amongst distinct cultures across the globe (Alves et al., 2014; Pandey et al., 2016; Landová et al., 2018; Onyishi et al., 2021), second, it can manifest itself very early in human ontogeny (Lobue and DeLoache, 2008; DeLoache and LoBue, 2009; Hayakawa et al., 2011; Borgi and Cirulli, 2015; Bertels et al., 2020), and third, we can observe a similar reaction in apes and other primates (Murray and King, 1973; Shibasaki and Kawai, 2009; Weiss et al., 2015; Kawai and Koda, 2016; Wombolt and Caine, 2016; Zhang et al., 2020). This body of evidence serves as the basis of what is now known as the Snake detection theory (Isbell, 2006 and references therein).

In the last two decades, the research of visual attention mainly focused on whether humans are able to detect a snake faster or more accurately than different types of stimuli in a challenging setup. In a visual detection task, snakes were detected faster than other animals (Lobue and DeLoache, 2008; Shibasaki and Kawai, 2011; Penkunas and Coss, 2013; Soares and Esteves, 2013), even under high perceptual load (Soares et al., 2014; Kawai and He, 2016; Kawai and Qiu, 2020) and regardless of their colouration (Prokop et al., 2018; Fančovičová et al., 2020). Similar results were obtained from a simulated virtual hike (Jensen and Caine, 2021), whilst LoBue (2014) and Kawai (2019) investigated which features facilitated the detection. Additional support for the Snake detection theory also comes from event-related potentials (ERP) studies (He et al., 2014; Grassini et al., 2016; Van Strien et al., 2016; Van Strien and Isbell, 2017) or neurobiological research (Van Le et al., 2013, 2014). Nonetheless, the issue seems more complex as, for example, guns (i.e., evolutionarily irrelevant inanimate objects) are detected as fast or even faster than snakes (Fox et al., 2007; Zsido et al., 2019a,b). Taken all together, there is a strong experimental support for primate brains being fine-tuned for snake detection, however, it seems that not all snakes are prioritised (see, e.g., Rádlová et al., 2019) and not under all circumstances (Subra et al., 2018; reviewed in Coelho et al., 2019).

Importantly, all these experiments assume – although sometimes inexplicitly – that humans and other primates pick on certain visual cues for snake detection provided by the snake’s appearance. It is worth pointing out that the snake does not provide these cues with the purpose of being detected; the primates rather take advantage of the cues the snake cannot conceal. In these scenarios, snakes are thought of as predators (Seligman, 1971; Öhman and Mineka, 2001; Isbell, 2006) and as such, they would not profit from being discovered. However, adult primates are rarely snake prey probably thanks to high vigilance, warning calls, and aggressive group defence (Seyfarth et al., 1980; Perry et al., 2003; Eberle and Kappeler, 2008; Etting et al., 2014; Teixeira et al., 2016). In fact, the roles might even reverse, and a snake might end up the prey itself (Headland and Greene, 2011; Falótico et al., 2018). What may have started as a clear predator–prey dynamics in evolutionary history, could now be seen rather as an equal-opponents situation.

Under these circumstances, it might be advantageous to signal one’s readiness to fight towards the opponent. This type of signalling is called a threat display. It is almost omnipresent in animals although it may take different forms in different species (e.g., chimpanzees – Nishida et al., 1999; frillneck lizards – Shine, 1990; cuttlefish – Langridge et al., 2007; pelicans – Gokula, 2011; tarantulas – Bennie et al., 2011). In snakes, the most famous example is the threatening posture of cobras – the animal puffs, its body front rises, and its neck-flack spreads (Greene, 1988). Another example comes from vipers – the animal puffs and coils its body in very tight loops with an elevated head held slightly back as if ready to strike (Greene, 1988). Both postures are quite conspicuous, and the animal often accompanies its display with hissing, which further facilitates its detection. The display is clearly intended to be seen by the opponent.

In this paper, we follow the line of thought previously introduced by Isbell’s Snake detection theory. We aim to explore whether the human mind is also fine-tuned for a snake’s intentional threat signalling rather than just unintentional cues of its presence. To this end, we employ an eye-tracking method utilising a simple design of spontaneous gaze preference when presented with two stimuli at once. We hypothesise that snakes in threatening postures will attract more attention than snakes in relaxed, non-threatening postures. In the past, it was demonstrated that emotions can guide visual attention (Vuilleumier, 2005), and, in particular, that fear-relevant animals are fixated faster, more often, or for longer time periods than fear-irrelevant animal targets (Öhman et al., 2001; Gerdes et al., 2009; LoBue, 2014). Moreover, the importance of snake posture for assessment of danger was previously shown in macaques (Etting and Isbell, 2014; Van Le et al., 2014), and humans (Masataka et al., 2010; Lobue and DeLoache, 2011).

To highlight the ecological aspect of our hypothesis, we focused on Somalis (specifically the population living in Somaliland). Whilst the culture is traditionally pastoral and therefore mobile, according to genetic and linguistic evidence they belong to the core populations of North and Northeast Africa, which have never left the African continent or the savanna environment. Somalis are thus characterised by the near-continuous presence in both the geographic region and the environment of human origin (Stringer, 2016; Gibbons, 2017). Moreover, evidence suggests that the snake species composition of the Horn of Africa has remained largely unchanged during the principal part of human evolution (Kelly et al., 2009; Barlow et al., 2019; Šmíd and Tolley, 2019; Zaher et al., 2019), and we previously found that Somalis consider snakes the most fear-eliciting animals amongst a wide variety of species (Frynta et al., 2023). This makes Somalis uniquely suited for research focusing on the possible co-evolution of snake signalling and human signal detection. In addition, we included participants from Czechia whose ancestors left Africa and, similarly to other Europeans, reached Europe about 30,000 years ago (Prüfer et al., 2021). As there have been virtually no dangerous snakes in Central Europe over the last 40,000 years (only mildly dangerous adder *Vipera berus*), Czechs seem a suitable match to Somalis for cross-cultural comparison. Similar responses across the participants, despite thousands of years of differential exposure to snakes, would suggest that the reaction is at least partially innate and a result of long coevolution between humans and snakes. Contrary, if cultural or more recent selection pressures are involved, clear differences between Somali and Czech participants should emerge. We know of no psychological study focusing on snakes and simultaneously utilising an eye-tracking experimental design in Sub-Saharan Africa.

To summarise, the aims of this study are as follows: (1) To test whether a snake in a threatening posture attracts more attention than one in a relaxed, non-threatening posture, (2) To investigate whether such phenomenon is universal or whether it is specific for certain snake morphotypes as those differ in their threatening postures, and (3) To compare the attention paid to snakes by Somalis and Czechs.

## 2. Materials and methods

### 2.1. Selection and preparation of the stimuli

The experimental stimuli were photos of 20 snake species. The selected snakes could be divided into three morphotype groups: vipers (eight species), cobras (eight species), and others (two pythons and two colubrid species). Vipers and cobras, all venomous species, were chosen because of their presumptive relevance for human evolution. During the selection process, the threatening posture of each candidate stimulus species was considered as it had to be visually distinctive enough from the relaxed body posture. This was an especially important criterion for the selection of non-venomous species which tend to have less conspicuous threat display. Lastly, we chose species distributed in Africa or the Middle East (except for two Asian species), the key regions of human evolution. Amongst the included snakes were also some of the most venomous species of the African Horn region: two vipers (puff adder *Bitis arietans* and North-East African Carpet Viper *Echis pyramidum*), two elapids (black mamba *Dendroaspis polylepis* and Egyptian cobra *Naja haje*), and one colubrid (boomslang *Dispholidus typus*). The Somaliland local fauna is additionally represented by two non-venomous species in our set (a colubrid species *Telescopus dhara* and a rock python *Python sebae*). Most stimuli photos came from authors’ personal archives but 15 were sourced from the internet. For a complete list of experimental stimuli and their sources, see Supplementary Table S1.

**Figure 1:**
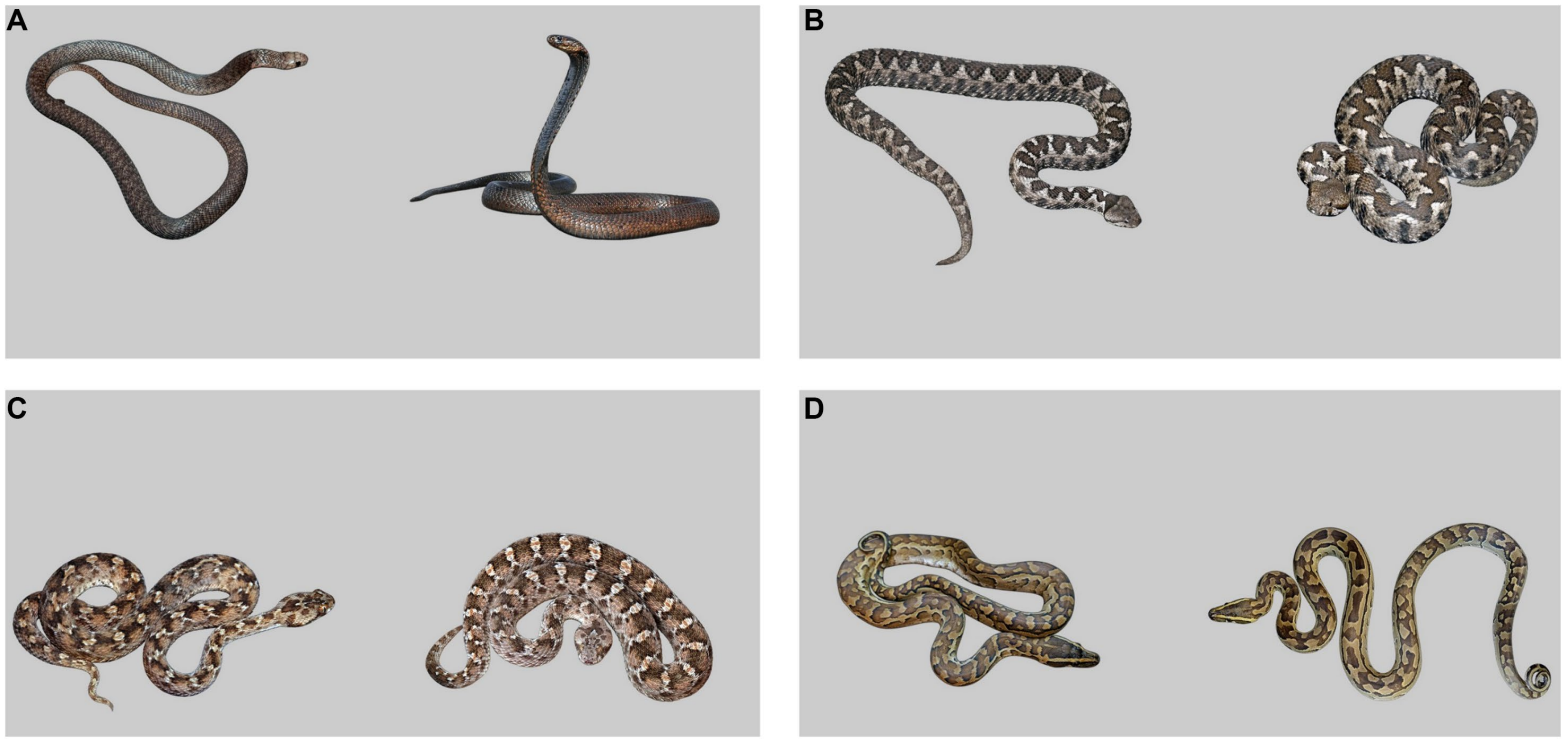
Example of experimental slides, the snake in threatening posture is always on the right. **(A)** Cobra morphotype, **(B–C)** viper morphotype, **(D)** other morphotype.

Each experimental image (slide) consisted of a photo of a snake in a threatening posture and a photo of the same specimen in a relaxed (non-threatening) posture. The original photos’ backgrounds were cut-off, and the snakes were placed on a shared 20% grey background, each on one side of the image. They were adjusted to be similar in size, hue, and brightness, and positioned so they both were looking towards the image centre. When available, pictures of the same snake individual were used. Twenty images with the threatening posture on the left were supplemented with their horizontally flipped versions (i.e., the threatening posture on the right) accounting for a total of 40 experimental images. In addition, one practise image preceded the experimental ones in the task. The practise image consisted of a drawing of a squirrel on the left and a hyena on the right. For examples of experimental images, see Figure 1.

### 2.2. The experimental procedure

Before the task itself, respondents were informed about the basic design of the experiment, and they gave written consent with their participation together with some personal information (name, gender, age, nationality). Next, participants were seated in front of the laptop (about 60 cm head to screen distance) with 1,366 × 768 pixels resolution, and they were asked to sit still but naturally and to look at the screen. This was followed by individual calibration of the myGaze eye tracking device. Immediately after a successful calibration, the experimental slide presentation followed. With the first slide (the practise image), they were instructed: “You will see two snakes at each slide, you may look at them as you wish, there is no particular task.”

The presentation consisted of 41 images (one practise image and forty experimental images), each displayed for 5 s. Between the images, there were slides with a black fixation cross on the same 20% grey background displayed for 2 s. The images were displayed in one of four pre-defined orders. Each order was a semi-random sequence of images where 10 stimuli (4 cobras, 4 vipers, 1 python, and 1 colubrid) were presented (both left and right versions) before the remaining 10 stimuli. The first and the second order were the same sequence of images, only displayed in reverse (the first image of order one was the last image of order two). The third and the fourth order were the same as order one and two, respectively, only the images were mirrored (left-versioned images were exchanged for right-versioned images and vice versa). Each respondent was assigned an order at random.

### 2.3. Participants

A total of 71 Somali and 71 Czech respondents participated in the experiment. In both samples, there were 25 women and 46 men. The mean age was 22.37 years (range 19–39) in the Somali sample and 24.63 years (range 18–44) in the Czech sample, the mean age did not significantly differ between the samples (non-parametric Man-Whitney test: Z-value = −1.26, value of *p* = 0.209). Most of the participants in both samples were undergraduate students of various fields. When asked by the investigator, no respondents expressed any extreme attitude towards snakes – neither positive (e.g., great fondness) nor negative (e.g., strong fear). The sample size was based on a similarly designed study (Rudolfová et al., 2022) where 136 participants in total were recommended by *a priori* power analysis using G*Power 3.1 (Faul et al., 2007). We chose a medium effect size (*f* = 0.15), adjusted the α error probability for multiple comparisons amongst categories (*p* = 0.0167), and corrected for a correlation amongst repeated measures (r = 0.25).

### 2.4. Data extraction and curation

MyGaze eye tracking device measures the position of the participants’ gaze and records approximately 30 samples per second. We developed our own processing software that converts the data into more intuitive variables which were defined as follows. “Number of sample measurements” is the total number of samples measured during the trial (i.e., approx. 150 in our case). A fixation was defined as all sample measurements that are no farther away than 23 pixels (0.5° visual angle) from a lead (reference) sample measurement. The lead sample measurement was defined as the first recorded measurement during each trial, and then each first measurement in the timeline that did not fall inside of the previous fixation (i.e., the next first measurement that was further them 23 pixels from the previous reference measurement). Moreover, each fixation had to consist of at least two consecutive sample measurements. Following these definitions, we computed the “Number of fixations.” Finally, “Fixation time” was defined as the total duration of the participant’s gaze. For the purpose of further analyses, we used only mean binocular metrics. Further, we custom-defined three interest areas (IAs) — the left side of the screen, the right side of the screen, and the central part (fixation cross) — and exported all variables separately for each IA. No IA overlapped any other. Left and right IAs covered the snake photos and their vicinity and were the same in size (each covering 37% of the screen). Central IA was very small in comparison (1% of the screen) and covered the area where the fixation cross would have been. Empty image parts too far from snake photos were not included in any of the IAs. To improve accuracy and precision, we used only averaged data from both eyes (Cui and Hondzinski, 2006).

For the subsequent statistical analysis, no participant was fully excluded, however, we eliminated defected measurements (trials) where the gaze was not tracked properly, or the participant was temporarily distracted and did not look at the screen. As a criterion, we chose to exclude observations with combined dwell time on the left and right AOIs under 2000 ms. Based on this criterion, 417 observations were excluded (7.34%). The final dataset, therefore, contained a total of 5,263 observations, 2,558 from Somali respondents and 2,705 from Czech respondents. To compensate for possible side preference, we averaged the data obtained from the horizontally flipped image pairs. In cases where only one slide of the pair was available (because the second one was excluded in the previous step), we used this data but assigned them observation weight “1.” Averaged data were assigned observation weight “2.” The original data associated with this manuscript are available in Supplementary Table S3.

### 2.5. Statistics

For the statistical analysis, we used linear mixed-effects models (LMM) as implemented in software R (R Development Core Team, 2022), packages nlme (Pinheiro et al., 2022), and emmeans (Lenth, 2022). Our prime focus was on the number of fixations supplemented by the analysis of dwell times (Orquin and Holmqvist, 2018). For investigation of the effect of threatening posture, we subtracted fixations on the snake in relaxed posture from fixations on the snake in threatening posture (always within the experimental slide) and thus prepared two new response variables – the difference in the number of fixations and the difference in the dwell time. We chose to investigate the difference rather than the absolute values on each posture because (1) it better reflected the pair manner of stimuli presentation and (2) represented specifically the effect of snake body position on participants’ spontaneous attention towards the stimuli. In full models, respondents’ gender, nationality, and age, and further group, gender-nationality interaction, and group-nationality interaction were used as fixed effects, whilst respondents’ ID was used as a random effect. To account for heteroscedasticity, we defined a custom variance structure combining the constant variance structure for respondents’ nationality and the fixed variance structure for observation weight. Fixed effects that did not prove significant (α = 0.05) were successively reduced. The reduced models and their respective full models were compared with the likelihood-ratio test and on the basis of the Akaike information criterion (AIC). The first method supported the same goodness of fit of both models (the full one and the reduced one), whilst the AIC suggested the reduced models were better because they were simpler (i.e., the full models were overfitted in comparison). Factor coefficients were computed using the restricted maximum likelihood method, for the purpose of full and reduced model comparison, we applied the maximum likelihood method.

### 2.6. Ethical note

All procedures performed in this study were carried out in accordance with the ethical standards of the appropriate institutional research committee (The Institutional Review Board of Charles University, Faculty of Science, approval no. 2019/2011, granted on 27 March 2019; and The Institutional Review Board of Amoud University, Borama, approval no. AU/AA/0012/2021, granted on 7 January 2021).

## 3. Results

**Figure 2:**
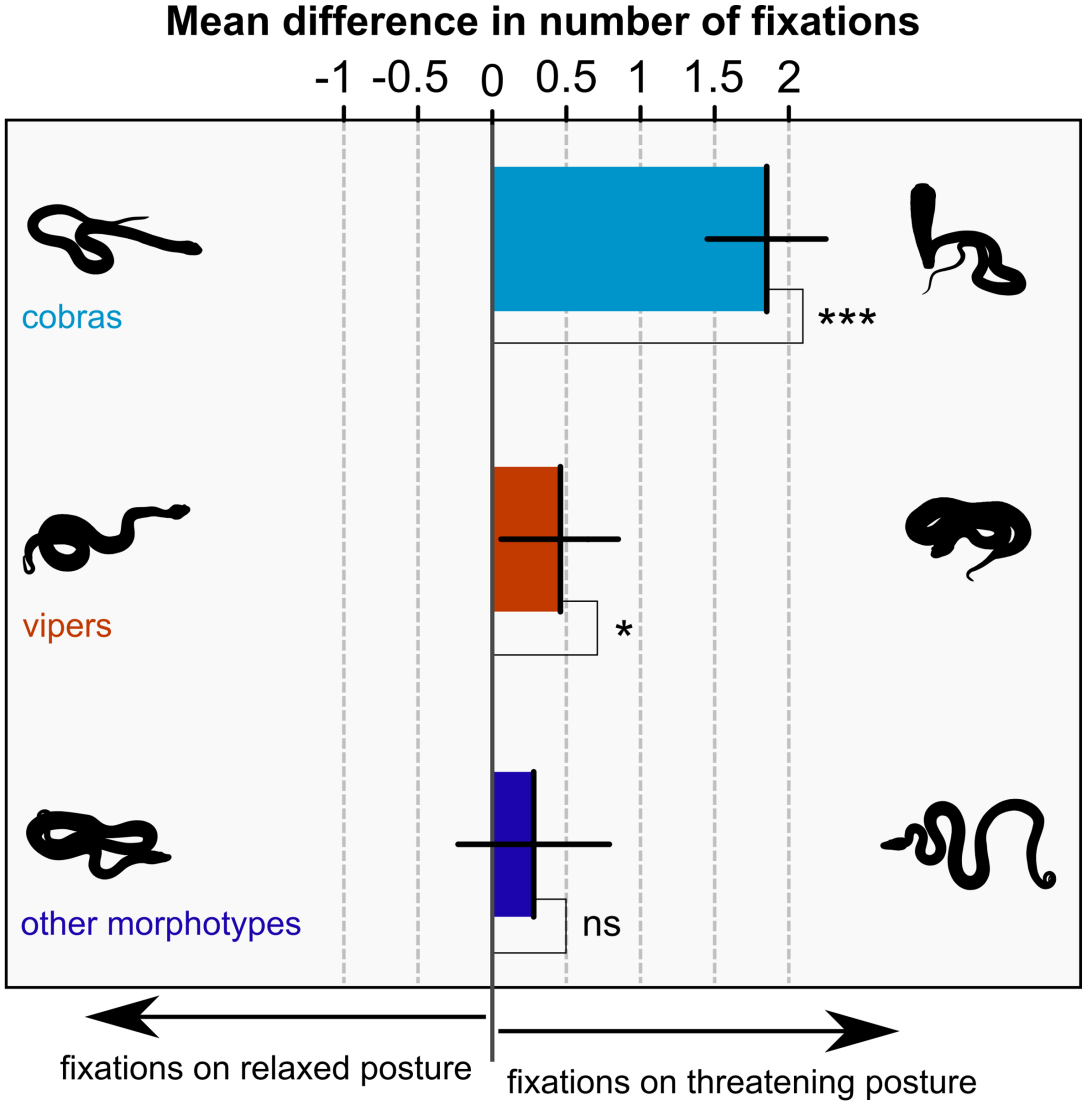
Mean difference of the number of fixations on snakes in the threatening versus relaxed posture for three snake morphotype groups. When the difference is positive, participants looked more at the threatening posture, when negative, participants looked more at the relaxed posture, zero difference signifies exactly equal attention on both. Error bars are 95% confidence intervals, means are tested against zero with significances indicated by asterisks (ns – *p* ≥ 0.05; * – *p* < 0.05; *** – *p* < 0.001).

In the linear mixed effect model for the difference in the number of fixations on threatening versus relaxed posture, only one factor proved significant: snake morphotype (*F*_(2,2,593)_ = 24.42, *p* < 0.001). Other factors were successively taken out of the model since their effect did not prove significant (nationality: *F*_(1,138)_ = 0.05, *p* = 0.830; gender: *F*_(1,138)_ = 1.54; *p* = 0.217; age: *F*_(1,138)_ = 0.20, *p* = 0.652; snake morphotype – nationality interaction: *F*_(2,2,591)_ = 2.26, *p* = 0.078; nationality – gender interaction: F_(1,138)_ = 0.05, *p* = 0.831). The odds ratio (assessed with likelihood-ratio test) between the full and reduced model was 7.00, *p* = 0.321. For the estimated differences, the following logic can be applied. When the difference is positive, participants looked more at the threatening posture, when negative, participants looked more at the relaxed posture, zero difference signifies equal attention on both. To this end, we tested each difference against zero. Cobras in threatening posture captured 1.85 more fixations than cobras in relaxed posture (95% confidence interval 1.45–2.25), this difference was significantly different from zero (*t*_(2593)_ = 9.12, *p* < 0.001). Similarly, vipers in threatening posture captured 0.46 more fixations than vipers in relaxed posture (95% confidence interval 0.06–0.85). Whilst the difference was smaller than the one for cobras, it was also significantly different from zero (*t*_(2593)_ = 2.28, *p* = 0.023). Lastly, other snake morphotypes captured 0.28 more fixations in threatening posture than in relaxed posture (95% confidence interval − 0.23 – 0.79), however, this difference was not significantly different from zero (*t*_(2593)_ = 1.09, *p* = 0.276). For context, the average number of fixations per trial across all stimuli and participants was 18.07 for the left and right IAs combined. The results are shown in Figure 2.

**Figure 3:**
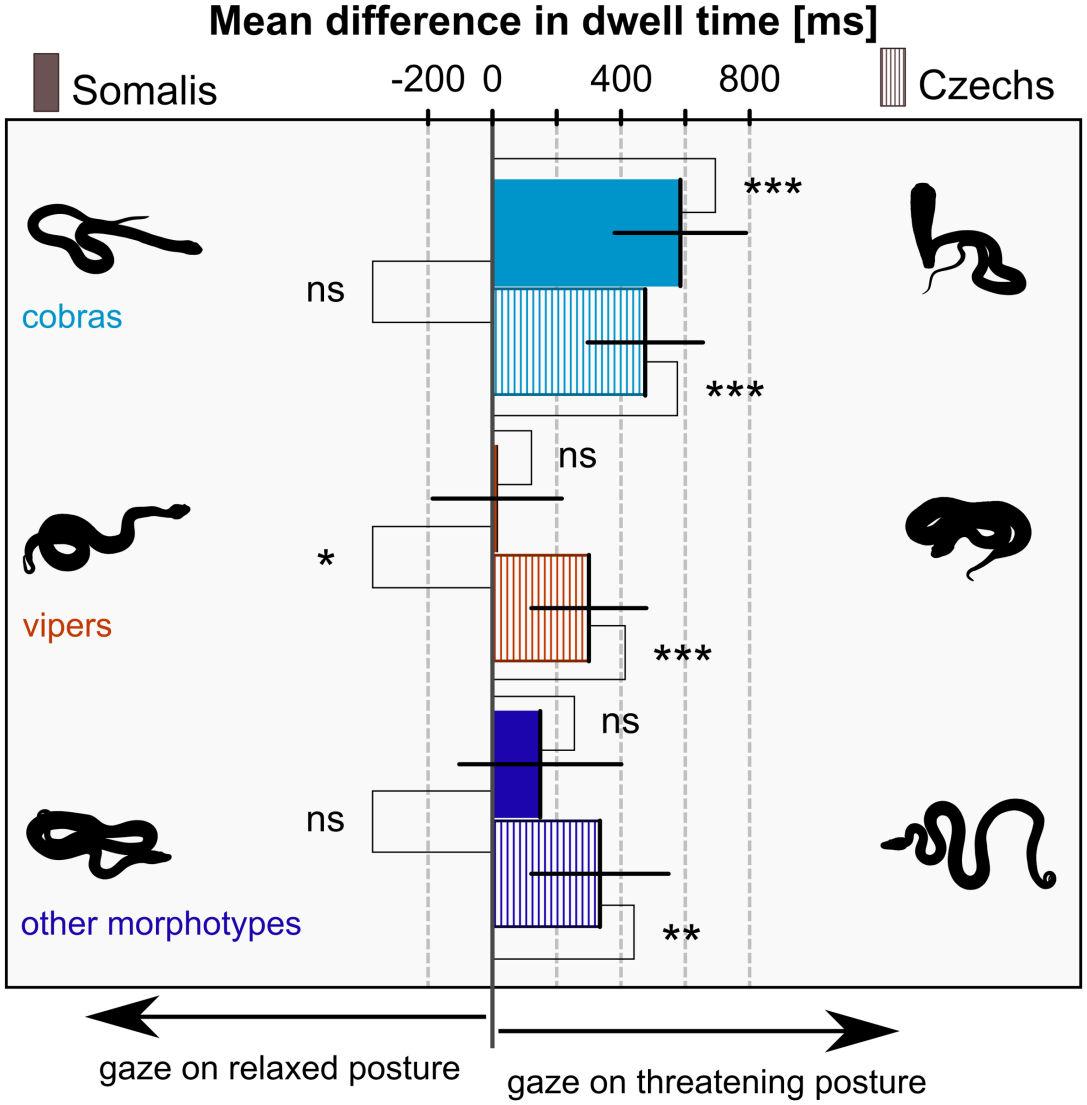
Mean difference of the dwell time on snakes in the threatening versus relaxed posture – the effect of snake morphotype and participants’ nationality. Positive values signify longer dwell time on the threatening posture, negative values on the relaxed posture, and zero signifies exactly equal attention on both. All means are tested against zero (right) and means of the same morphotype are compared between Somalis and Czechs (left). Error Bars are 95% confidence intervals, significances are indicated by asterisks (ns – *p* ≥ 0.05; * – *p* < 0.05; ** – *p* < 0.01; *** – *p* < 0.001).

There was no difference in the goodness of fit of the full and reduced model for the difference in dwell time (*p* = 0.300), hence we again chose the reduced model as the final model based on the AIC. The odds ratio between the models was 3.66. The final model for the difference of dwell time on threatening versus relaxed posture contained snake morphotype (*F*_(2,2,591)_ = 12.57, *p* < 0.001), nationality (*F*_(1,141)_ = 0.94, *p* = 0.333), and their interaction (F_(2,2,591)_ = 4.33, *p* = 0.013). Other factors were successively taken out of the model (gender: *F*_(1,138)_ = 3.15; p = 0.078; age: *F*_(1,138)_ = 0.50, *p* = 0.504; nationality – gender interaction: *F*_(1,138)_ < 0.01, *p* = 0.975). The results showed that both Somalis and Czechs gazed at cobras in a threatening posture longer than at cobras in a relaxed posture. In viper and other snake morphotype stimuli, only Czechs dwelled on the snakes in threatening posture longer; Somalis divided their attention equally. For more details, see Table 1 and Figure 3. The average dwell time per trial across all stimuli and participants was 4,368 ms for the left and right IAs combined. For average dwell times per trial of each stimulus species, see Supplementary Table S2.

**Table 1:** Results of the model for the difference of dwell time on threatening versus relaxed posture.

Difference of dwell time (threat. – relax. posture)		Estimate	95% CI	df	*t*-value	Value of *p*
Somalis	Cobras	584.7	380–789	2,591	5.65	**<0.001**
	Vipers	15.4	−186 – 216	2,591	0.15	0.879
	Other morphotypes	148.8	−103 – 401	2,591	1.17	0.243
Czechs	Cobras	475.3	296–655	2,591	5.24	**<0.001**
	Vipers	300.2	121–479	2,591	3.32	**<0.001**
	Other morphotypes	334.5	121–548	2,591	3.10	**0.002**
Somalis vs. Czechs	Cobras	109.4	−162.7 – 381.4	141	0.79	0.428
	Vipers	−284.7	−553.7 – −15.7	141	−2.09	**0.038**
	Other morphotypes	−185.7	−515.9 – 144.5	141	−1.11	0.268

The first six estimates are tested against zero – positive values signify longer dwell time on the threatening posture, negative values on the relaxed posture, and a zero estimate signifies exactly equal attention on both. The estimated means and 95% confidence intervals (95% CI) are in milliseconds. Value of *ps* <0.05 are in bold. Df stands for degrees of freedom.

## 4. Discussion

To summarise the results, we found that snakes in a threatening posture attracted more attention than those in a relaxed, non-threatening posture which was manifested both in the number of fixations and the total dwell time. This was especially prominent in cobras, however, vipers in a threatening posture were also fixated more often than vipers in a non-threatening posture. Additionally, differences in dwell time revealed that Czechs dwelled on snakes in threatening posture longer no matter the snake morphotype. Contrarily, in Somalis, this held true only for the cobra morphotype; in the other two investigated morphotype groups (vipers and others) there was no difference in the attention paid to the snakes in threatening and non-threatening postures. Nonetheless, the only significant difference between Somalis and Czechs was in their reaction to vipers and specifically only in the dwell time, not the fixation count. Hence, we consider the overall pattern of reaction towards snake threatening postures relatively consistent across nationalities. We found no effect of gender.

In the last couple of years, researchers paid special attention to what features are crucial for the recognition of a snake. An early candidate in question was a curvilinear body shape. Whilst this feature can play an important role (Wolfe et al., 1992; LoBue, 2014), alone, it is most probably not sufficient as worms (of a similar curvilinear body shape) evoked a smaller reaction in an ERP study (Van Strien et al., 2016). Next, the effect of body posture was investigated. Lobue and DeLoache (2011) suggested that a snake’s coiled body shape is responsible for the faster detection of snakes in relation to distractors. However, Masataka et al. (2010) argued that the striking (vs. relaxed) body posture rather enhanced the speed of detection but was not its basis *per se*. This notion was supported by Etting and Isbell (2014) in their rhesus macaque behavioural study. Recently, the topic of recognition-relevant features of snakes was reviewed by Kawai (2019). In concordance with the most current results, he concluded that snake scales are the key characteristic (He et al., 2014; Van Strien et al., 2016; Isbell and Etting, 2017; Van Strien and Isbell, 2017; Kawai, 2019). Interestingly, the importance of snake scales could be illustrated already in hominids, as some palaeolithic engravings likely represent a snakeskin (Coss and Charles, 2021). Although we agree with the studies on the importance of snake scales, this feature cannot explain the results of this study since it is the key for snake identification only in the context of other animals. Therefore, we propose to take a step back and look at the bigger picture once more.

Above, we suggested that the primate visual system is not only adapted for faster detection of snakes (Isbell, 2006, 2009) but also fine-tuned for the snake’s threat display. Notably, some snake-typical features which were previously found to enhance the speed of snake detection seem to be exaggerated in snakes’ threat display. In the case of vipers, such feature is the coiled shape (Lobue and DeLoache, 2011; Etting and Isbell, 2014), which is very tight under the threat creating many loops, whilst in cobras, the risen body front exaggerates the snake’s curvilinear body shape (Wolfe et al., 1992; LoBue, 2014). These exaggerated features typical for threat display might be behind the larger spontaneous attention paid to snakes in threatening postures. Moreover, the erect posture (seen in threatening cobras) is very conspicuous, and many animal species take advantage of it when wanting to intimidate an opponent. Indeed, people perceive animals in an erect posture as more fear-eliciting (Prokop et al., 2021). This, however, opens a question of whether the attentional privilege of snakes in threatening posture is driven by visual or affective features. It has been previously shown that emotions can modulate attention towards a stimulus. Soares et al. (2009) reported that participants found the animal they were afraid of faster than non-feared but fear-relevant animals. Similar results were also found by Miltner et al. (2004) or Lipp and Waters (2007). The arousal might also affect visual attentional performance (reviewed in Zsido, 2023). For instance, Zsido et al. (2022) showed that stimulus arousal might be an important cue facilitating target recognition in a memory test. As for now, however, we do not have enough data to decide the extant visual and affective features of snakes in threatening posture modulate attention and it might even be that these two types of features are inseparable in nature.

Although the overall pattern of reaction towards snakes was similar for both Somalis and Czechs, we would also like to shortly address the found difference. Czechs dwelled longer at the snake in threatening posture no matter the morphotype, but the same was true only for cobras in Somalis. Could it be that Somalis differentiate (in a perceptual sense) between threatening and non-threatening postures only in cobras simply because the difference is the most conspicuous? This explanation would not fit well into the evolutionary framework and indeed, it is not the case. In a complementary study, individual photos of snakes very similar to photos used in this experiment were presented to Somalis and they were asked to order them according to elicited fear from the most to the least fear-eliciting (Frynta et al., in prep.). In this forced-choice experiment, six out of eight viper species were rated as significantly more fear-eliciting when in the threatening posture than in the non-threatening posture (Frynta et al., in prep.). We instead suggest that Somalis attribute the same level of threat to vipers no matter their body posture leading to the same observed dwell time. LoBue (2014) previously showed that knowledge or expectations can interact with low-level features of the stimuli in visual search tasks. In our case, the (communal) knowledge of the nature of cobra and viper attacks seems to be the key. Cobras are active in their defence; they either flee or display to the opponent and strike only afterwards. Vipers, on the other hand, are passive; they often rely on their cryptic coloration and motionless stance and strike when the opponent (usually unknowingly) comes too close. Even though the majority of Somali participants were university students now living in a city, most of them came from rural areas and pastoral families. We find it very likely that they personally knew someone that was bitten by a snake. When we asked local villagers, at least one person was willing to share their experiences in every village. No communal knowledge can be expected in Czechs since local snake fauna is not dangerous to humans and, moreover, participants were mostly from urban areas. This finding might be of importance for future studies since it illustrates that not all snakes are the same (see also Landová et al., 2018, 2020; Janovcová et al., 2019; Rádlová et al., 2019; Frynta et al., this issue).

To conclude, our results show that human attention is directed more towards cobras and also vipers in threatening postures. We hypothesise that it is a result of primate-snake coevolution during which not only snakes represented a danger to primates but also primates represented a danger to snakes. To be clear, we do not argue that snakes evolved specific threatening postures in response to predator pressure from primates. That is very unlikely because of multiple reasons including primates are not snake’s primary predators, and the threatening postures are not addressed uniquely to primates but to a variety of other potential predators, e.g., mongooses (Herpestidae) or birds (the secretarybird *Sagittarius serpentarius*, crested seriema *Cariama cristata*, or others). We simply argue that at some point in evolutionary history, the primate-snake relationship must have become less one-sided since this is the situation we witness today (Headland and Greene, 2011; Falótico et al., 2018; see also Harris et al., 2021). As a part of their defensive behaviour, snakes would be displaying threat and these threat signals would in turn become associated with intense danger leading to prioritised attention toward threatening displays. The features of the threat display could not be reliably used for the detection of snakes because the threatening posture is a defensive behaviour and hence is not displayed when hunting for prey. Nonetheless, prioritised attention is still adaptive as the threat display signals the immediate danger of a ready-to-strike snake. Although the prioritised attention towards snakes was previously tested in several different cultures, this is the first study investigating a population from Sub-Saharan Africa – a key region with regards to the evolutionary Snake detection theory.

## Data availability statement

The original contributions presented in the study are included in the article/Supplementary Material, further inquiries can be directed to the corresponding author.

## Ethics statement

The studies involving humans were approved by The Institutional Review Board of Charles University, Faculty of Science and The Institutional Review Board of Amoud University. The studies were conducted in accordance with the local legislation and institutional requirements. The participants provided their written informed consent to participate in this study.

## Author contributions

DF and EL: conceptualization. IŠ: data curation. IŠ and DF: formal analysis, writing – original draft, and writing – review & editing. DF: funding acquisition and supervision. IŠ, MJ, VR, HE, KR, DK, DS, DB, PF, and DF: investigation. All authors contributed to the article and approved the submitted version.
